# Spectral Overlap Limits Optical Gain in Quantum Heterostructure Photonic Devices

**DOI:** 10.1002/nap2.70230

**Published:** 2026-07-28

**Authors:** Nathalie Lander Gower, Muna Abedelrahman, Shiran Levy, Mohammed Salih, Maor Engel, Sumona Kumar, Sadhvikas J. Addamane, A. Giles Davies, Edmund H. Linfield, Asaf Albo

**Affiliations:** ^1^ Faculty of Engineering and Institute of Nanotechnology and Advanced Materials Bar‐Ilan University Ramat Gan Israel; ^2^ Department of Electrical and Electronic Engineering Ariel University Ariel Israel; ^3^ School of Electronic and Electrical Engineering University of Leeds Leeds UK; ^4^ Center for Integrated Nanotechnologies Sandia National Laboratories Albuquerque New Mexico USA

## Abstract

Population inversion in quantum heterostructures underpins light generation in a wide range of photonic devices, including quantum cascade lasers and intersubband emitters. It is commonly assumed that increasing carrier density enhances inversion and optical gain; however, this trend can reverse at elevated densities due to many‐body interactions. Here, we investigate this behavior using a controlled doping series in terahertz quantum cascade lasers combined with self‐consistent nonequilibrium Green's function simulations. We show that Coulomb‐induced quasiparticle broadening increases with carrier density and competes directly with the intrinsic energy separation of the active states, reducing the spectral selectivity of optical transitions. We introduce a dimensionless spectral‐overlap parameter, Γ_avg_/Δ, where Γ_avg_ represents the interaction‐induced optical linewidth and Δ is the interlevel energy separation, and demonstrate that gain across distinct device architectures follows a common trend with a maximum near Γ_avg_/Δ ≈ 1. Beyond this point, spectral overlap suppresses inversion and limits optical gain. These results reveal a density‐driven crossover in which many‐body broadening sets a fundamental spectral limit on optical gain in quantum‐confined photonic systems. The Γ_avg_/Δ parameter provides a universal design metric for identifying inversion limits and optimizing performance in quantum heterostructure light‐emitting devices.

## Graphical abstract

Increasing carrier density is commonly expected to enhance optical gain. Here, a universal crossover is identified in which interaction‐induced spectral broadening competes with the intrinsic energy separation of optical states. When Γ_avg_/Δ approaches unity, spectral overlap suppresses transition selectivity and limits gain, revealing a fundamental design constraint for quantum‐confined photonic devices.

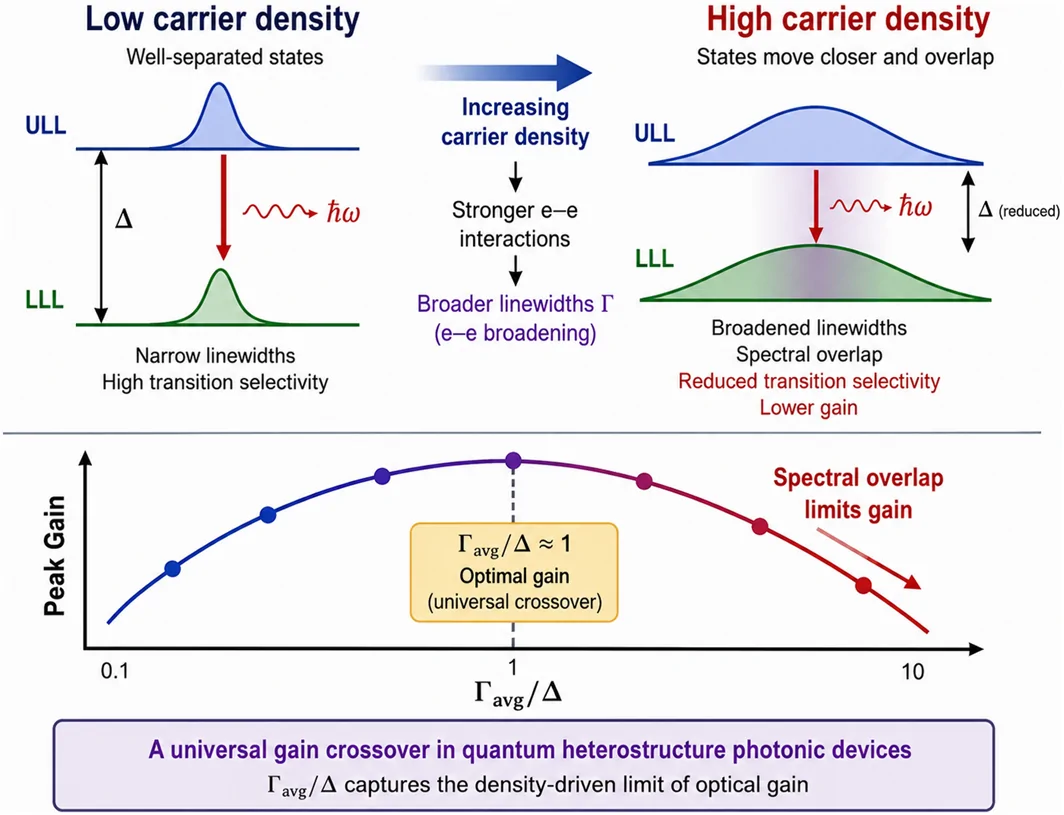

## 1 Introduction

Population inversion in quantum heterostructures underpins light generation in a wide range of photonic devices, including quantum cascade lasers and intersubband emitters [1, 2, 3, 4, 5, 6, 7, 8]. More broadly, optical gain in quantum‐confined systems relies on spectral selectivity between discrete states, a requirement that becomes increasingly fragile in the presence of many‐body interactions. As carrier density increases, interaction‐induced broadening can compromise the spectral distinction between transitions, fundamentally limiting the efficiency of light generation. Understanding this interplay is, therefore, essential not only for quantum cascade lasers but also for a wide class of quantum‐engineered photonic devices. It is commonly assumed that increasing carrier density enhances inversion and optical gain by strengthening population imbalance between relevant quantum states [1, 2, 3, 9, 10]. However, at elevated densities, many‐body interactions introduce additional physical mechanisms that can fundamentally alter this behavior [11, 12, 13, 14, 15, 16, 17, 18].

In particular, carrier–carrier interactions lead to quasiparticle broadening of energy levels, increasing the spectral linewidth of optical transitions [14, 19, 20, 21]. When this broadening becomes comparable to the intrinsic energy separation between active states, the resulting spectral overlap reduces transition selectivity and can suppress population inversion. Despite its fundamental nature, this interplay between interaction‐induced broadening and level spacing has not been formulated as a general criterion governing inversion in quantum‐engineered heterostructures.

Here, we identify a density‐driven crossover governed by a dimensionless spectral‐overlap parameter, Γ_avg_/Δ, where Γ_avg_ is the interaction‐induced linewidth and Δ is the interlevel energy separation. Using a combination of experimental measurements and self‐consistent nonequilibrium Green's function simulations [19, 22, 23, 24, 25, 26, 27, 28, 29, 30, 31, 32, 33, 34, 35], we show that optical gain across distinct device architectures collapses onto a similar curve with a maximum near Γ_avg_/Δ ≈ 1. This establishes a general spectral constraint on optical gain, revealing a regime in which many‐body broadening governs optical response and limits transition selectivity in quantum‐confined photonic systems.

Here, we isolate this density‐driven many‐body contribution using nonequilibrium Green's function (NEGF) simulations of TWI‐DP architectures, where impurity and parasitic leakage channels are strongly suppressed [36, 37, 38, 39, 40], thereby providing a representative platform to probe the general spectral‐overlap mechanism. By systematically varying doping while maintaining alignment bias, we identify a crossover regime where Coulomb‐induced linewidth broadening becomes comparable to the upper–lower laser level separation. Expressing the peak gain in terms of the spectral‐overlap parameter $\Gamma_{\text{avg}}/\Delta$, we show that inversion saturates and subsequently decreases once many‐body broadening approaches the level spacing, revealing a density‐driven crossover governing gain behavior. This behavior reflects a general spectral crossover that arises when interaction‐induced quasiparticle broadening becomes comparable to the intrinsic energy spacing of the optical transition. In this regime, spectral selectivity of the transition breaks down, and the concept of well‐resolved lasing states ceases to apply.

## 2 Device Platform

Using a controlled series of TWI‐DP devices with identical layer sequences but varied carrier densities, together with NEGF calculations, we isolate electron–electron (e–e) scattering as the mechanism limiting gain under optimized transport conditions. The NEGF calculations are performed in a real‐space basis along the growth direction, whereas Wannier–Stark states are used as a convenient representation for visualization. The devices exhibit a pronounced negative differential resistance (NDR) region persisting to room temperature [41], indicating suppressed leakage and transport confined primarily to the designed active subbands. Setback doping further reduces dopant overlap with the lasing states, minimizing impurity‐induced broadening.

The TWI‐DP architecture (Figure 1) confines transport to four principal subbands while keeping higher‐lying levels energetically remote. An injection doublet couples resonantly to the upper laser level (ULL) of the adjacent period, whereas the lower laser level (LLL) aligns with the injection states of the next period, enabling efficient phonon‐assisted depopulation [7, 39, 42, 43, 44]. The diagonal lasing transition reduces ULL–LLL wavefunction overlap and suppresses LO‐phonon scattering [9, 10, 36, 45, 46]. With LO‐phonon and impurity contributions strongly suppressed, the remaining intrinsic density‐dependent mechanism is e–e scattering.

**FIGURE 1:**
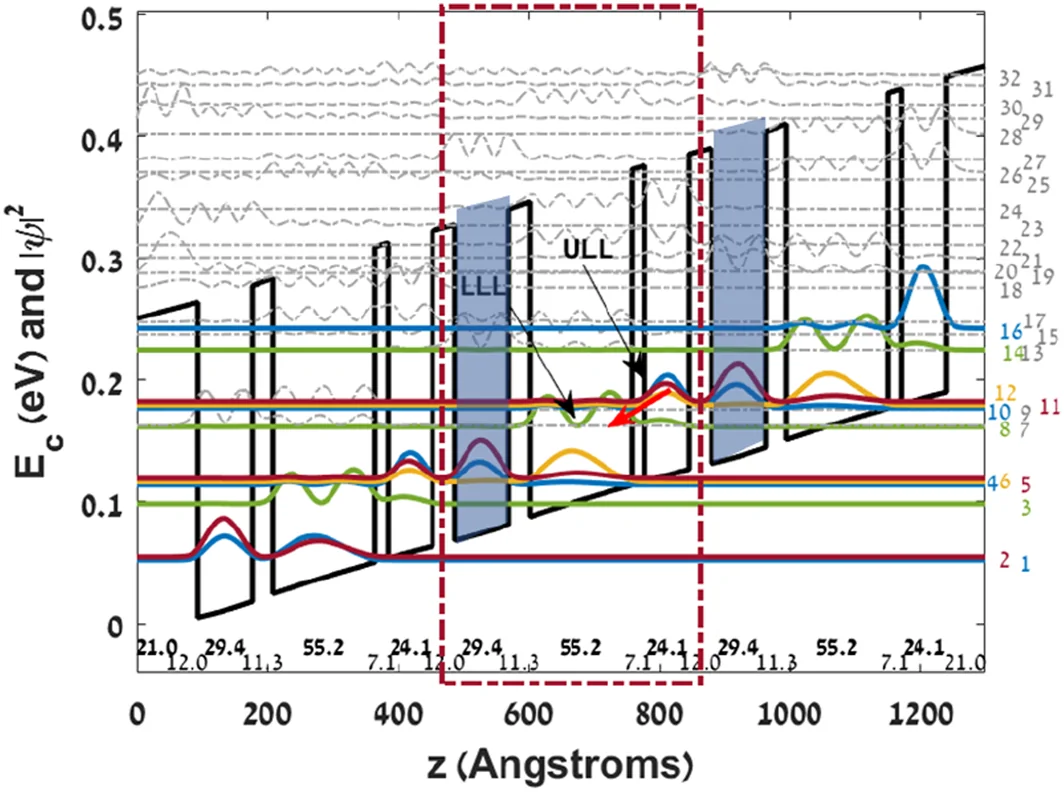
Schematic band diagram of the two‐well injector direct‐phonon (TWI‐DP) THz QCL design [41]. The upper (ULL, blue) and lower (LLL, green) laser levels are indicated, together with the setback‐doped region. The injection doublet (dark red–yellow) couples resonantly to the ULL, whereas the diagonal lasing transition (red arrow) and spatial separation of dopants suppress LO‐phonon and impurity scattering, respectively, enabling isolation of density‐driven many‐body effects. Full layer parameters and device details are provided in Supporting Information S1.

## 3 Results

A series of structures spanning nominal doping densities of $6\times 10^{10}$–$1.2\times 10^{11}$ cm^−2^ were grown in a single MBE campaign and processed identically, isolating carrier‐density variation as the only controlled parameter. All devices lase near 4 THz and exhibit transport consistent with the designed subband scheme. Temperature performance remains moderate and does not improve monotonically with increased doping (Figure 2), deviating from simple population scaling.

**FIGURE 2:**
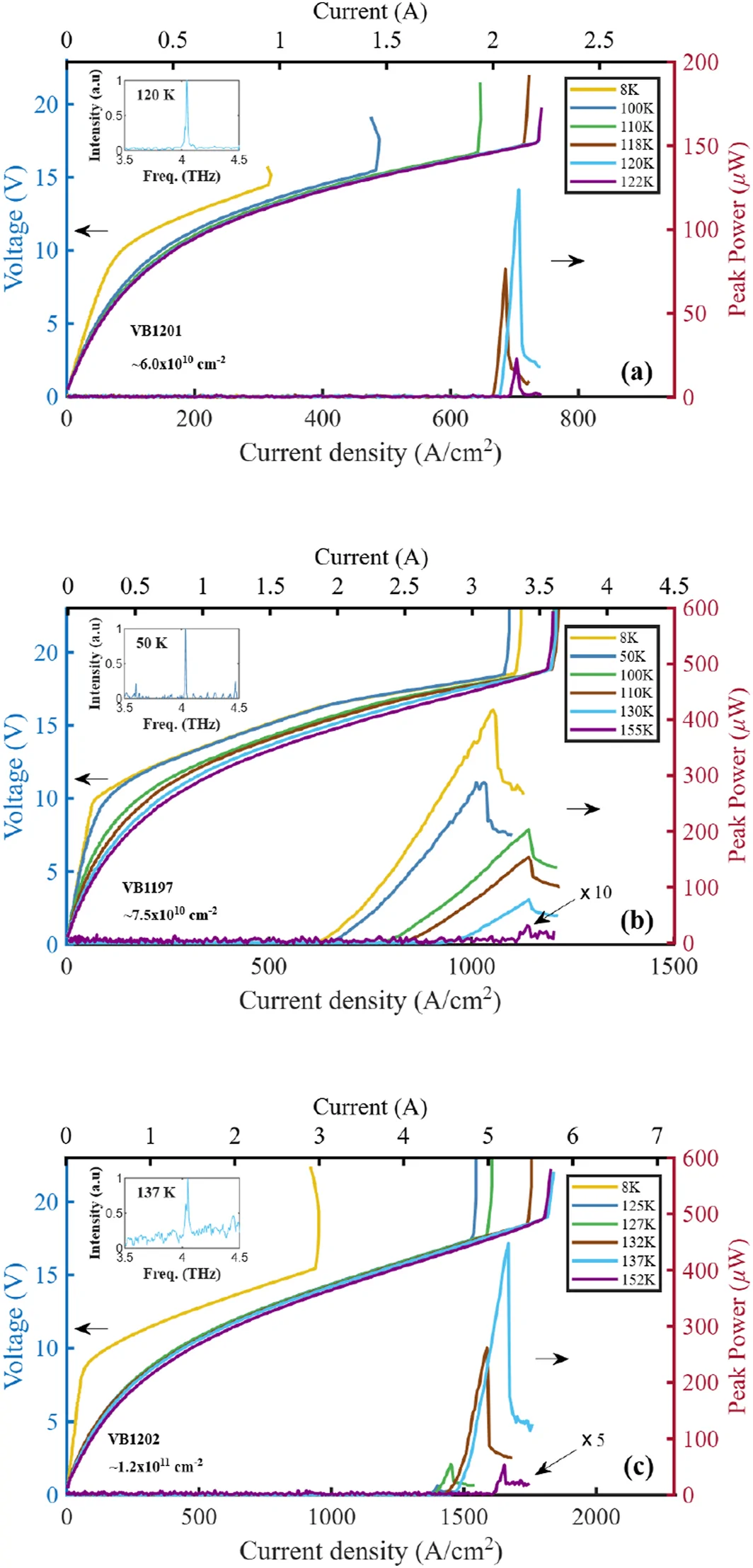
Pulsed light–current (L–I) and voltage–current (V–I) characteristics measured at various temperatures for three TWI‐DP structures with different doping densities (a, b, c). All devices lase near 4 THz, as shown in the emission spectra (insets). The weak additional spectral features in the spectra insets are attributed to measurement noise; only a single lasing mode is observed near 4 THz. For VB1201/VB1202, early negative differential resistance (NDR) suppresses lasing at low temperatures; however, lasing occurs in a higher‐temperature regime after recovery. Hence, temperature performance is not affected by this feature. Temperature performance remains moderate and does not improve with increased doping. Device parameters and measurement details are provided in Supporting Information S1.

NEGF simulations reproduce this trend (SM). Although all devices exceed typical metal–metal waveguide losses (∼15 cm^−1^ [47], consistent with our calibrated loss values at the lasing frequency) at low temperature, peak gain decreases with increasing doping even at 10 K. Simulated gain–temperature characteristics show that the lowest‐doped structure maintains inversion over the broadest range, whereas higher‐doped devices undergo earlier gain collapse.

Because LO‐phonon scattering is suppressed by the diagonal transition and impurity scattering is minimized by setback doping [41], neither mechanism explains the degradation. Selective removal of scattering channels within NEGF (Figure 3 and Supporting Information S1) shows that eliminating interface roughness or impurity scattering produces only modest gain changes, whereas removing e–e scattering yields substantial enhancements—up to ∼150%, ∼200%, and ∼300% with increasing doping. These results identify density‐dependent e–e interactions as the dominant limiting mechanism under optimized transport conditions.

**FIGURE 3:**
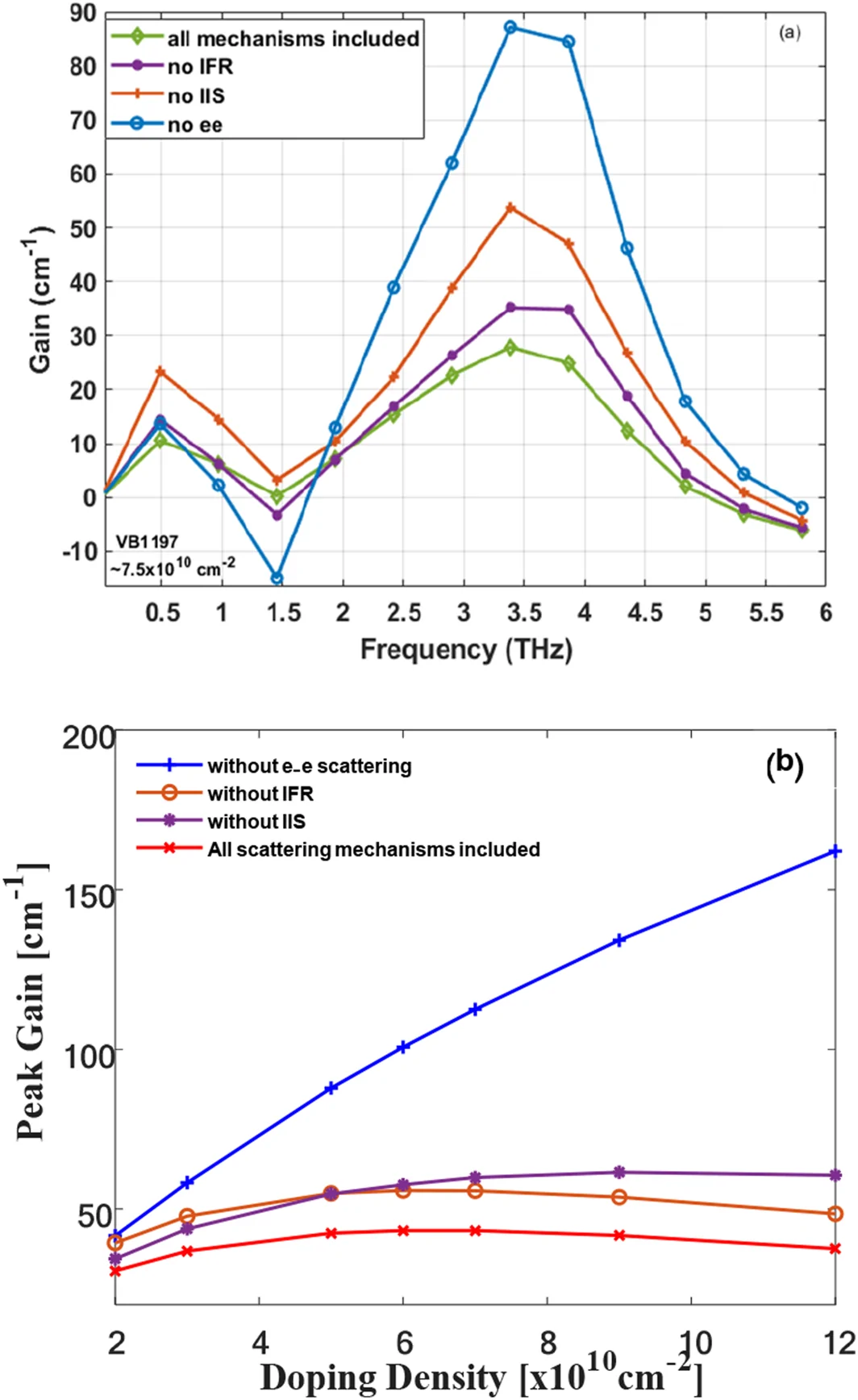
(a) NEGF‐calculated optical gain at 10 K for the middle‐doped TWI‐DP structure, illustrating the effect of selectively removing individual scattering mechanisms. Suppressing interface roughness (IFR) or impurity‐induced scattering (IIS) produces minor changes, whereas removing electron–electron (e–e) interactions results in a pronounced gain increase. (b) Peak gain versus sheet doping from self‐consistent NEGF simulations, comparing the full model with channel‐elimination cases. When all scattering mechanisms are included, peak gain saturates with increasing doping; removing e–e scattering restores a monotonic increase. Device parameters are provided in Supporting Information S1.

Figure 3b shows that peak gain saturates with increasing doping when all scattering mechanisms are included, whereas removing e–e scattering restores a monotonic gain increase. This behavior reflects the density‐driven many‐body crossover governed by interaction‐induced spectral broadening. The same trend is reproduced in the previously published split‐well resonant‐phonon (SWRP) platform (Supporting Information S1) [48].

Spectral functions of the ULL (Figure 4a) clarify the origin of this trend. Suppressing impurity scattering produces only minor linewidth narrowing, consistent with reduced dopant–subband overlap, whereas suppressing e–e scattering yields a substantially sharper spectral function that becomes weakly dependent on doping. The direct correlation between linewidth broadening and gain degradation identifies e–e scattering as the dominant intrinsic mechanism under these optimized conditions. The effect strengthens with carrier density and is not eliminated by setback doping or similar strategies.

**FIGURE 4:**
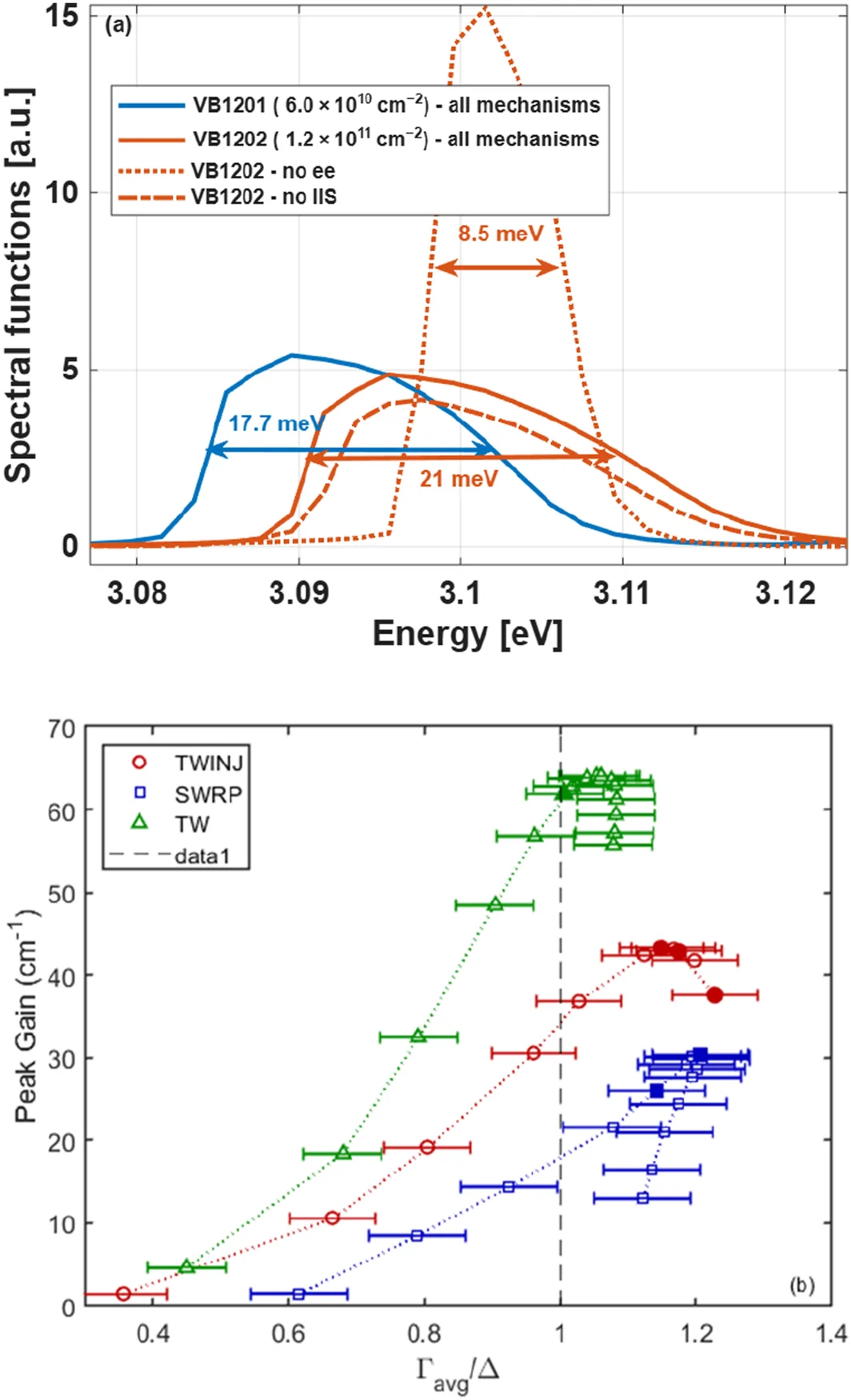
(a) NEGF‐calculated spectral functions of the upper laser level (ULL) for lower‐ and higher‐doped TWI‐DP structures with all scattering mechanisms included (solid lines). For the higher‐doped structure, calculations are also shown without electron–electron (e–e) interactions (dotted) and without ionized‐impurity scattering (IIS, dot‐dashed). Removing e–e scattering results in substantial linewidth narrowing compared to suppressing impurity scattering. Full‐width‐at‐half‐maximum (FWHM) values are indicated for the two structures with all mechanisms included. (b) Peak material gain as a function of the spectral‐overlap parameter Γ_avg_/Δ, where Γ_avg_ = (FWHM_ULL_ + FWHM_LLL_)/2 and Δ is the ULL–LLL energy separation evaluated at the alignment bias (63 mV for TWI‐DP and 55 mV for SWRP). The two‐well (TW) structure G938 from Ref. [8] is also included for comparison. Dotted lines are included to indicate the trends. Here, Γ_avg_ represents the effective optical linewidth arising from many‐body interactions in the lasing states. All data are computed from the corresponding spectral functions. Filled markers denote experimentally realized doping values. Horizontal error bars reflect the ± 1.4 meV uncertainty in linewidth extraction. Data from all three architectures collapse onto a similar trend, indicating that optical gain in quantum‐confined emitters is governed by a single spectral scaling parameter Γ_avg_/Δ, independent of device architecture. Gain increases as Γ_avg_ → Δ, reaches a maximum near Γ_avg_/Δ ≈ 1, and decreases for Γ_avg_/Δ > 1, marking the onset of spectral‐overlap‐induced inversion degradation. The vertical dashed line indicates the crossover condition Γ_avg_ ≈ Δ, where interaction‐induced broadening becomes comparable to the intrinsic level spacing. Although this crossover marks the onset of spectral overlap, the exact position of the maximum gain remains somewhat dependent on the specific design details.

Notably, the many‐body linewidth Γ_avg_ can approach the ULL–LLL separation Δ, marking a crossover where the lasing transition is no longer spectrally isolated. Lasing can persist in this regime because gain depends on both population inversion and spectral overlap; however, increasing broadening progressively reduces transition selectivity and net gain.

To isolate the governing parameter, we express peak gain in terms of the spectral‐overlap parameter $\Gamma_{\text{avg}}/\Delta$, where $\Gamma_{\text{avg}}=\left({\text{FWHM}}_{\text{ULL}}+{\text{FWHM}}_{\text{LLL}}\right)/2$ and Δ is the ULL–LLL separation at the alignment bias of each structure. The linewidths include all scattering mechanisms within NEGF. However, they are dominated by e–e scattering, so $\Gamma_{\text{avg}}$ primarily reflects doping‐induced broadening. As shown in Figure 4b, both TWI‐DP and SWRP collapse onto a similar curve, indicating that gain is governed primarily by the dimensionless ratio Γ_avg_/Δ rather than device‐specific details. The experimentally realized doping values lie within this regime, consistent with operation beyond the onset of overlap‐induced gain saturation. The collapse across distinct injector architectures indicates that the relevant control parameter is the ratio of many‐body linewidth to level separation, establishing a broadly applicable crossover condition independent of specific device design. More generally, this demonstrates that Γ_avg_/Δ acts as a universal control parameter for inversion in driven quantum heterostructures. An additional curve for the two‐well (TW) structure from Ref. [8], which holds the record temperature performance to date, is also shown in Figure 4b for reference. It exhibits the same crossover behavior, with peak gain occurring near $\Gamma_{\text{avg}}/\Delta \approx 1$.

Earlier studies of SWDP and SWRP designs [18, 20, 40, 48, 49, 50] attributed the absence of doping‐induced performance improvement to combined impurity and e–e scattering. In contrast, the TWI‐DP platform suppresses impurity and LO‐phonon pathways, enabling isolation of density‐driven Coulomb broadening. Our combined experimental and theoretical results show that increasing doping strengthens e–e interactions and accelerates gain collapse with temperature. Accordingly, strategies such as setback doping that narrow impurity‐induced linewidths in absorption spectroscopy do not mitigate e–e broadening in operating QCLs. Once the device enters the e–e‐limited regime, doping optimization alone cannot improve high‐temperature performance.

This behavior reflects a fundamental trade‐off between carrier density and spectral selectivity that is intrinsic to quantum‐confined optical transitions.

## 4 Conclusions

These findings identify a density‐driven crossover in quantum heterostructures that emerges once Coulomb‐induced spectral broadening becomes comparable to the energy separation between active states. Expressing the gain in terms of the spectral‐overlap parameter Γ_avg_/Δ reveals that peak inversion occurs near Γ_avg_/Δ ∼ 1, beyond which increasing carrier density reduces spectral selectivity and suppresses optical gain.

Although the TWI‐DP architecture provides a controlled platform in which parasitic leakage and impurity mechanisms are strongly suppressed, the observed behavior demonstrates that increasing doping alone cannot improve performance once the system enters this interaction‐dominated regime. The consistent crossover behavior observed across three distinct injector architectures further indicates that the governing parameter is the ratio of many‐body linewidth to level separation rather than device‐specific structural details.

More broadly, the Γ_avg_/Δ ratio acts as a universal spectral control parameter for inversion in quantum‐confined photonic systems. This dimensionless criterion establishes a simple and physically transparent design rule for identifying the onset of inversion degradation and optimizing performance in quantum‐engineered light‐emitting devices.

## Author Contributions

**Nathalie Lander Gower:** investigation, writing – original draft, formal analysis, data curation. **Muna Abedelrahman:** investigation, formal analysis, data curation. **Shiran Levy:** investigation, data curation, formal analysis. **Mohammed Salih:** investigation, data curation, formal analysis. **Maor Engel:** investigation, project administration. **Sumona Kumar:** investigation, data curation. **Sadhvikas J. Addamane:** investigation, resources. **A. Giles Davies:** investigation, funding acquisition, supervision, resources. **Edmund H. Linfield:** investigation, funding acquisition, supervision, resources. **Asaf Albo:** conceptualization, investigation, funding acquisition, supervision, project administration, writing – review and editing, resources, formal analysis, writing – original draft, methodology, validation.

## Supporting information


Supporting Information S1

## Data Availability

The data that support the findings of this study are available from the corresponding author upon reasonable request.
