# Supplementary material for: Spectral Overlap Limits Optical Gain in Quantum Heterostructure Photonic Devices
Source: Nanophotonics. 2026 Jul 28;15(15):e70230. doi: 10.1002/nap2.70230 (PMC13642156; doi:10.1002/nap2.70230)
Supplement: Supplementary file 1 [file NAP2-15-e70230-s001.docx]

Supplementary Material

**Additional NEGF Analysis of TWI-DP Structures**

**(b)**

**(a)**

Fig. S1: NEGF simulation results of: (a) Gain at 10 K temperature as a function of frequency at the bias voltage for each device, and (b) Peak gain as a function of temperature at the bias voltage for each device. Losses were estimated to be ~ 15 cm−^1^ (horizontal line).

Fig. S2: NEGF simulation results of the spectral functions of the ULL (as a representative level) of VB1201 (blue), VB1197 (orange), and VB1202 (yellow), including all the scattering mechanisms (solid line), without e-e scattering (dotted line) and without IIS (dot-dashed line). The FWHM is shown here only for VB1197 with all scattering mechanisms and without e-e scattering for reference; further details can be found in the main manuscript for other devices.

**SWRP -NEGF calculations**

Fig. S3: Peak gain versus sheet doping from self-consistent NEGF simulations of the SWRP structure [24], comparing the full model including all scattering mechanisms with channel-elimination cases (including without e–e). The results show a gain ceiling with increasing doping when all mechanisms are included, while removing e–e restores a monotonic gain increase. Full device parameters are provided in Refs. 24, 30, 72.

**More details about the NEGF**

In the NEGF simulations, electron–electron (e–e) scattering is included using the self-energy formalism, which accounts for both intrasubband and intersubband Coulomb interactions. The interaction is modelled by (i) a Hartree mean-field term within the electrostatic Poisson equation, and (ii) an incoherent scattering term, treated in an elastic and static approximation with a Coulomb scatterer distribution that follows self-consistently the electron density. This implementation reproduces the essential effects of e–e scattering—carrier energy redistribution, level broadening, and dephasing—allowing close agreement with experiment. The simulations also provide subband electron temperatures, which show strong non-equilibrium heating at low lattice temperatures and convergence toward the lattice value at higher temperatures, consistent with previous reports. More details on the simulation can be found in Refs. 57, 58, 59.

**Experimental details**

For L-I-V measurements the QCL was operated in pulsed mode using 400 ns pulses at a repetition rate of 500 Hz (0.02% duty cycle). A cryocooler bolometer with a responsivity of 15 kV/W was used for detecting the signal.

The THz emission spectra were measured using a Bruker IFS 66 Fourier Transform Infrared (FTIR) spectrometer operated in rapid-scan mode with a spectral resolution of 7.5 GHz. The emitted THz beam was collimated and focused into the spectrometer using off-axis parabolic mirrors. Spectra were recorded using a liquid-helium-cooled bolometer (Infrared Laboratories) with a responsivity of 89.6 kV/W mounted externally to the FTIR spectrometer. The FTIR spectrometer was purged with dry nitrogen. The spectrum was obtained after applying a 3-point Blackman-Harris apodization function.

**Device and design parameters of the TWI devices in this study**

Table S1: Main nominal design parameters and device data.

| **Device** | **Lasing Energy [meV]** | **E_21_ [meV]** | **Oscillator Strength** | **Nominal Expected Activation Energy [meV]** | **E_6-13_**  **[meV]** | **Layer Sequence [Ǻ], barrier composition and doping level** | **Process Details** |
| --- | --- | --- | --- | --- | --- | --- | --- |
| Device 1 (VB1201) | 14.5 | 43.7 | 0.56 | 21.5 | 103.9 | **34**/84/**32**/156/**20**/68  254 periods  Total thickness 10 µm  GaAs/**Al_0.3_Ga_0.7_As**  7.1×10^16^ cm^-3^ in the 84 Å well  (6.0×10^10^ cm^-2^). | metal-metal  (100 Å Ta / 2500 Å Au)  Top contact n^+^ layer was removed, bottom contact is 50 nm thick GaAs with doping of 5×10^18^ cm^-3^  Dry Etched  Mesa size 150 µm × 2 mm |
| Device 2 (VB1197) | 15.1 | 43.9 | 0.55 | 20.9 | 104.8 | **34**/83/**32**/156/**20**/68  255 periods  Total thickness 10 µm  GaAs/**Al_0.3_Ga_0.7_As**  9.1×10^16^ cm^-3^ in the 83 Å well  (7.6×10^10^ cm^-2^). |  |
| Device 3 (VB1202) | 14.0 | 43.7 | 0.56 | 22.0 | 107.2 | **34**/81/**32**/156/**20**/68  256 periods  Total thickness 10 µm  GaAs/**Al_0.3_Ga_0.7_As**  1.5×10^17^ cm^-3^ in the 81 Å well  (1.2×10^11^ cm^-2^). |  |

| **Device**  **(Wafer)** | **Design electric field**  **[kV/cm]** | **τ^0^_ul_ [ps]*** | **τ^0^_21_ [ps]**** | **Lasing Energy [meV]** | **Expected Activation Energy [meV]** | **Experimental T_max_ [K]** |
| --- | --- | --- | --- | --- | --- | --- |
| Device 1 (VB1201)  (doping density ∼6.0 × 10^10^ cm^−2^) | 15.7 | 0.65 | 0.61 | 16.7 | 19.3 | 122 |
| Device 2 (VB1197)  (doping density ∼7.5 × 10^10^ cm^−2^) | 16.1 | 0.67 | 0.83 | 16.7 | 19.3 | 155 |
| Device 3 (VB1202)  (doping density ∼1.2 × 10^11^ cm^−2^) | 15.8 | 0.64 | 0.55 | 16.7 | 19.3 | 152 |

Table S2: Device parameters and performances.

*ULL to LLL raw LO-phonon scattering time, determined from simulation.

**LLL (level 2) to Injector (level 1) LO-phonon scattering time, determined from simulation.
